# Variability in Abundance of Temperate Reef Fishes Estimated by Visual Census

**DOI:** 10.1371/journal.pone.0061072

**Published:** 2013-04-04

**Authors:** Alejo J. Irigoyen, David E. Galván, Leonardo A. Venerus, Ana M. Parma

**Affiliations:** Centro Nacional Patagónico–Consejo Nacional de Investigaciones Científicas y Técnicas, Puerto Madryn, Chubut, Argentina; Leibniz Center for Tropical Marine Ecology, Germany

## Abstract

Identifying sources of sampling variation and quantifying their magnitude is critical to the interpretation of ecological field data. Yet, most monitoring programs of reef fish populations based on underwater visual censuses (UVC) consider only a few of the factors that may influence fish counts, such as the diver or census methodology. Recent studies, however, have drawn attention to a broader range of processes that introduce variability at different temporal scales. This study analyzes the magnitude of different sources of variation in UVCs of temperate reef fishes off Patagonia (Argentina). The variability associated with time-of-day, tidal state, and time elapsed between censuses (minutes, days, weeks and months) was quantified for censuses conducted on the five most conspicuous and common species: *Pinguipes brasilianus*, *Pseudopercis semifasciata, Sebastes oculatus*, *Acanthistius patachonicus* and *Nemadactylus bergi*. Variance components corresponding to spatial heterogeneity and to the different temporal scales were estimated using nested random models. The levels of variability estimated for the different species were related to their life history attributes and behavior. Neither time-of-day nor tidal state had a significant effect on counts, except for the influence of tide on *P. brasilianus*. Spatial heterogeneity was the dominant source of variance in all but one species. Among the temporal scales, the intra-annual variation was the highest component for most species due to marked seasonal fluctuations in abundance, followed by the weekly and the instantaneous variation; the daily component was not significant. The variability between censuses conducted at different tidal levels and time-of-day was similar in magnitude to the instantaneous variation, reinforcing the conclusion that stochastic variation at very short time scales is non-negligible and should be taken into account in the design of monitoring programs and experiments. The present study provides baseline information to design and interpret results from visual census programs in temperate reefs.

## Introduction

Accounting for or minimizing “unexplained” sampling variability represents a challenge in all fields of ecology, given its importance for correctly interpreting field data [1]–[5]. Most monitoring programs of reef fish populations aim to assess spatial and/or temporal changes in their abundance; for example, to assess the effects of fishing by comparing protected to unprotected areas. Indices of abundance of reef fishes collected through such programs are affected by multiple sources of variability, whose magnitude depends on the characteristics of the habitats and species monitored. Despite the importance of understanding the sources of variability for experimental design and data analysis, surprisingly few studies have quantified the variability associated with different temporal scales and processes, which may introduce “noise” in the data and potentially confound the results.

Different methods are used to collect field data for estimating reef-fish abundance (e.g., gillnets, trapping, video recording and visual censuses, see [6]–[13]). Among them, underwater visual census (UVC) is the most commonly used in shallow waters because it offers several advantages: censuses are non-destructive sampling techniques, therefore suitable for use in marine protected areas (MPAs) and on long-lived rare species; they work for a wide range of fish sizes and behaviors; and they are cost-effective in terms of money, time and logistics, compared to video recording. In addition, divers may register several variables and gather direct observations while they count fish (e.g., Reef Life Survey Program, URL: http:\\www.reeflifesurvey.com). Drawbacks of UVC techniques, however, are biases in the estimates of abundance of cryptic species and small fishes, problems with recounting mobile individuals, lack of a permanent record, and diver avoidance of some species [10], [11], [14]–[17].

Sources of variation in visual census datasets were classified by Thompson & Mapstone [4] in three general categories: (1) real changes in abundances due to recruitment and/or loss of individuals to populations; (2) temporary local shifts in the distribution of individuals, without net change in population size; and (3) sampling error. Sampling error is composed of systematic error (due to low detectability of some species or fishes) and random factors (e.g., counting errors), both influenced by the diver and directly related to his/her experience [14]–[16]. Detectability is defined as the probability of observing a particular species during a given sampling occasion conditional on its presence at that location [18]. UVC-based studies routinely assume that detectability equals one for all species, although in reality it may vary substantially among reef fishes and habitats in relation to behavioral and life-history characteristics. For example, estimation bias may be substantial in the case of cryptic or small species [17], [19], [20]. The random portion of the sampling error is composed of simple counting error plus real variation in local abundance introduced by random displacements of fish across the boundaries of the sampling units, at very short time scales (e.g., seconds to minutes). Both contribute to what McClanahan et al. [14] called “instantaneous variation”, which they found to be a large source of variation for the coral reef fishes they surveyed, often overlooked in previous works (i.e., which attributed it to larger temporal scales). Counts of fast swimming and schooling species are particularly affected by this source of variation. Aside from diver effects, which can be readily estimated [19], the remaining sources of instantaneous variability in fish counts are difficult to discriminate as they all contribute to base sampling error [14], [16], [21]. At larger temporal scales, displacements in response to tidal level or time-of-day (e.g., in response to light) may result in increased variation at the scale of hours, while seasonal migrations associated with changes in water temperature or reproductive cycles would increase variation at the scale of months. The assessment of actual changes in abundance over longer time periods (often the scale of interest) resulting from trends in recruitment and mortality, or from shifts in distribution (e.g., shifts in latitudinal range associated with climate change), needs to take into account the magnitude of all sources of variability. However, most studies based on UVC data typically consider only a few such sources (e.g., diver effect, time-of-day) [22]–[24], and methods used to partition variability amongst the different components, including not only temporal but also spatial variation, may be inappropriate if they fail to account for the different sources. The investigation of sources of error and variability in UVC is a rising and fast-developing field due to its relevance for a correct interpretation of the data and for the design of appropriate monitoring programs (e.g., [4], [14], [16], [17], [21]–[25]).

The aim of this study was to assess the magnitude of temporal sources of variation in UVCs conducted on a low-diversity reef-fish assemblage, typical of temperate shallow reefs in the northern Atlantic Patagonian coast. Two types of variation were considered: (1) differences in time-of-day and tidal level, treated as deterministic factors, and (2) those associated with different temporal scales, treated as stochastic factors. A unified statistical framework based on nested random-effect models was used to analyze the data, while accounting for spatial heterogeneity between reefs. The paper presents an original case study from temperate waters, which provides information relevant for survey design and interpretation of past UVC data used for monitoring temperate reef systems.

## Materials and Methods

### Northern Patagonia rocky reefs and fishes

In the study area, reefs are formed by isolated small rocky outcrops that extend for a few hundred meters on an otherwise flat, soft bottom. These reefs are mainly linear structures, typically breaks or ledges (up to 4 m high and 6 m wide) located along the edge of submerged abrasion limestone platforms, where cavities are formed. Crumbled portions of the ledges determine width and structural complexity of the reefs.

The northern Patagonia reef-fish assemblage has a low species diversity: it is composed of 29 species belonging to 21 families [26], but only five of species (four families) are conspicuous and commonly found in the reefs: *Pinguipes brasilianus*, *Pseudopercis semifasciata, Sebastes oculatus*, *Acanthistius patachonicus* (see [27] for a taxonomic update for this species) and *Nemadactylus bergi* [26]. This work focuses on these most common non-cryptic species, which are easily detectable by standard strip transect visual censuses. While *N. bergi* is a schooling species, the rest are sedentary demersal fishes, strongly associated to refuges [26].

### Study site

Fish censuses were conducted at 12 shallow rocky reefs near Punta Pardelas (42.7°S 64.3°W), Nuevo Gulf, from March 2007 to February 2008. This site was selected because of the large number of reefs and good visibility (between 5 m and 10 m). Because the abundance of *P. semifasciata* in Pardelas was too low to provide reliable estimates of variability, data from UVCs conducted in San José Gulf (42.3°S 64.4°W) were used (see [24] for details), corresponding to three shallow rocky reefs censused between July 2002 and May 2004 (reefs B, C and E in [24]). Those reefs were similar in shape, topography and depth to the reefs from Pardelas, and the visual census technique used was comparable (see below).

The reefs selected at Nuevo Gulf were small ledges (24 m to 150 m in length by up to 5 m in width), separated from each other by 50 m to 1.2 km. Reefs from San José Gulf were much farther apart (from 3.7 km to 19.6 km), up to 3 m wide, and between 52 m and 315 m in length. Reefs depth ranged between 5 m and 16 m at low tide. The tidal regime is semidiurnal and the mean tidal amplitude is 3.8 m in Nuevo Gulf and 5.7 in San Jose Gulf, but spring tides could reach 5.7 m and 8.8 m, respectively.

### Underwater visual census methods

Censuses of the 12 reefs selected in Nuevo Gulf were all conducted by the same diver, by swimming along a fixed 25 m×5 m strip transect along the reef ledge. A single transect was randomly positioned in each reef at the beginning of the study, and delimited by iron pickets driven permanently into the bottom. Three sequential passes were made of each transect, swimming at a constant speed (24 m/min), to count individuals of A. patachonius, S. oculatus and P. brasilianus, in that order, using standard methodology [4], [16], [24], [28]. To increase the probability of detection of the schooling species N. bergi, whose counts involved at most one single school, this species was counted on each pass and the maximum number encountered recorded.

At San José Gulf, the entire reefs were censused by three divers, who counted all *P. semifasciata* observed while swimming along the ledges. Three replicated censuses were completed on each sampling occasion, one by each diver in random order, waiting 5–10 minutes between successive censuses. No significant effects of order of census (i.e., diver disturbance) nor diver identity were detected when those effects were evaluated as fixed factors using these data [24].

Because fish are only present on a narrow band (generally less than 3 m wide) along the reef ledges, and given the good visibility conditions in the area, divers were able to survey the whole width of the reefs. Divers swam continuously, only counting fishes larger than 10 cm of total length in order to avoid possible biases associated with small size categories (e.g., [4], [17]). They also avoided counting fish that appeared from behind.

### Sampling design

The reefs were censused at controlled tidal levels (low vs. high tide) and times of day (morning vs. afternoon), and at different time periods (from minutes to months), in order to quantify the variability in fish counts associated with the different factors and temporal scales. The censuses conducted in Nuevo Gulf to evaluate short-term effects were concentrated in the fall, when water temperature reaches a maximum of ∼17°C (minimum temperature occurs in August and is approximately 9°C).

In order to evaluate the effects of tidal state and time-of-day on fish counts, sampling dates and times were selected so that each factor was allowed to vary one at a time while keeping the other factor constant (for further details see Text S1). These deterministic factors were evaluated separately and prior to the onset of the remaining temporal censuses; this approach was similar to that used by Thompson & Mapstone [4]. Once the effect of time-of-day was found to be non-significant for all species (see Results), the effect of tidal state was evaluated by surveying all transects at low and high tides during the same day. Tidal amplitude on the sampling day (5 May 2007) was 4.3 m, whilst it ranged between 3 m and 4.5 m over the study period.

**Figure 1:**
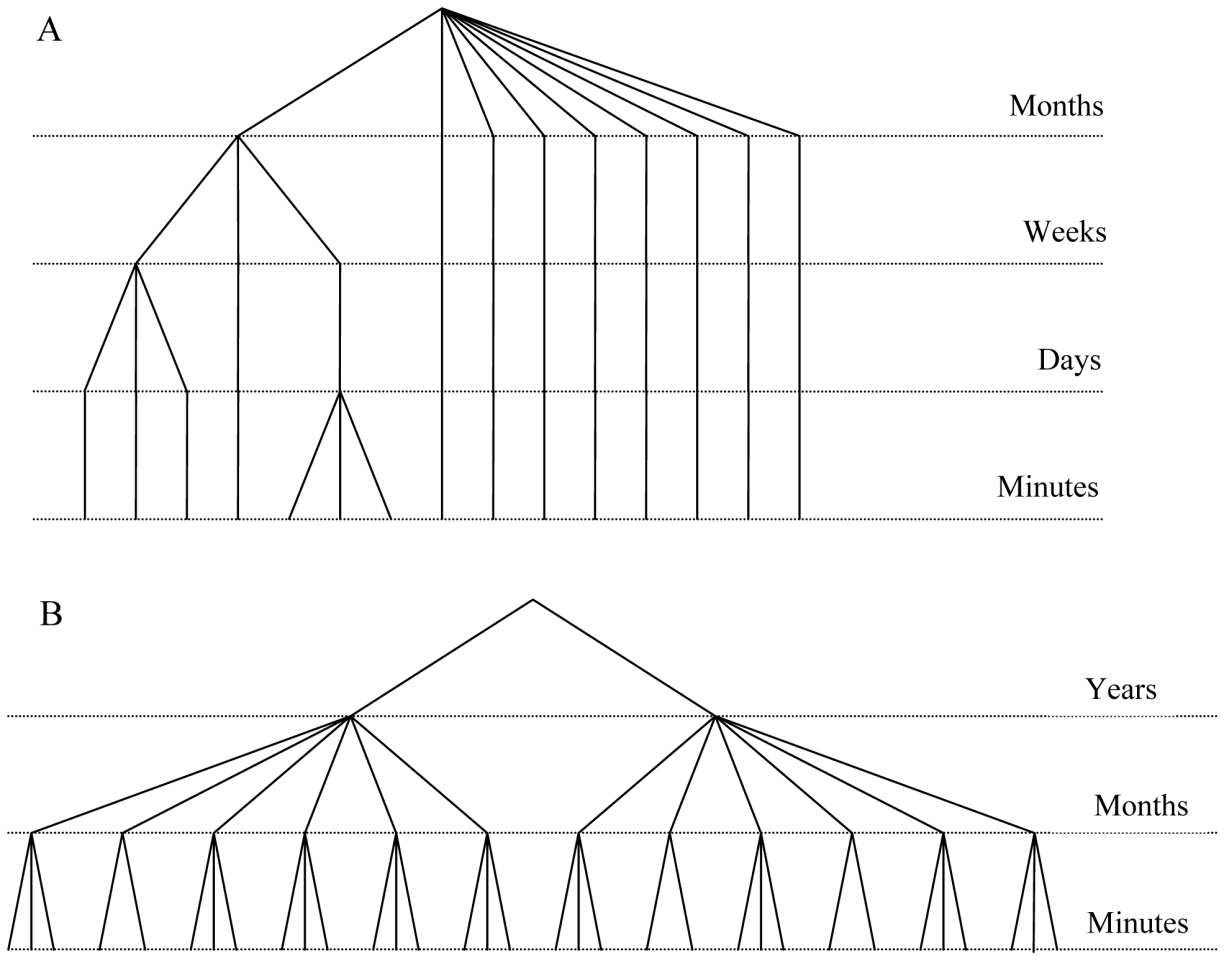
Sampling design used to evaluate variability at different temporal scales. Scheme of the sampling design followed on each reef, for each of the five species censused in 12 reefs within Nuevo Gulf (A), and for *Pseudopercis semifasciata* censused in three reefs from San José Gulf (B).

To estimate instantaneous variation, three replicated counts were made for each species on each of the 12 transects, waiting 5 minutes between successive series (each series consisted of three sequential passes, one per species as explained earlier). These censuses were done on 4 April 2007 (transects 1 to 9) and on 4 May 2007 (transects 10 to 12) (Fig. 1). To estimate day-to-day (i.e., daily) variation in fish counts, all transects were surveyed once a day during three consecutive days (between 0900 hrs and 1300 hrs), on 19–21 March 2007. The initial date was selected opportunistically depending on logistics and weather conditions, and the initial census was delayed ∼45 min per day in order for the tidal height to remain approximately constant. Variability at the scale of weeks was assessed based on censuses conducted on 19–21 March 2007, 29 March 2007, and 4 April 2007 (Fig. 1).

Finally, to estimate intra-annual variation, all transects were surveyed on an approximately monthly basis (*n* = 9), between 0900 hrs and 1300 hrs, from March 2007 to February 2008, covering an annual cycle. Sampling dates did not follow a strictly regular schedule, but were dependent on weather; thus tidal height varied among censuses.

Censuses of *P. semifasciata* in San José Gulf were conducted over a two-year period to investigate the species' temporal pattern of reef occupancy [24]; hence that data set only allowed assessment of instantaneous and intra-annual variability in counts, but not the effects of tidal height, time-of-day, and daily and weekly variation (Fig. 1). These latter sources of variability therefore contribute to the intra-annual variability estimated.

### Statistical analysis

The variability of fish counts associated with different factors and temporal scales was estimated separately for each species using log-linear mixed and random-effect models. The models were fitted by restricted maximum likelihood (REML) using the *lme4* and *nlme* libraries [29], [30] implemented in the R software [31]. REML was preferred to maximum likelihood (ML) because it compensates for the downward bias of the ML estimates of variance components [29], [32] and it is the recommended methodology for estimating variance components in unbalanced designs [32], [33]. In all cases (mixed- and random-effect models), the transect identity was treated as a random effect in order to account for variation associated with spatial heterogeneity, i.e., heterogeneity among the experimental units given by reef depth, reef geomorphology, bottom type, availability of refuges, etc. Time-of-day and tidal state were evaluated as fixed factors, with levels: morning and afternoon, and low and high tide, respectively. Their significance was assessed by a *t*-test, conditional on the estimates of the random effects variance parameters [29].

In order to quantify the variability contributed by the different temporal scales, a nested random-effect model was fitted, which included day, week and month as random factors, all nested within transect (see Text S2). All random effects were assumed to be normally distributed with mean zero and variances 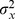, where *x* represents a given random effect. The variance estimated for each of the random effects (spatial and temporal) measures the contribution of each source to the total variability. The residual error was interpreted as the base sampling error or instantaneous variability.

The percent contribution of each source to the total variance was calculated from the ratio of each variance to the sum of all variance components. The significance of each random component was evaluated by likelihood ratio tests [29], comparing the full model with one in which the given component had been dropped, one at a time. Additionally, the nested model for *P. semifasciata* included year as a random factor given that the data were collected over a two-year period. Note that the standard deviation (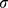) estimated with log-transformed data is close to the coefficient of variation (*CV*) in the natural scale, as 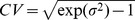. The *CV*s corresponding to variation due to spatial heterogeneity among reefs, 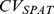, and temporal variation added at increasing temporal scales,
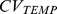
(*TEMP*: instantaneous, day, week, month) were calculated from the respective estimated variances. Confidence bounds (95%) for each standard deviation were calculated using the function *intervals* of the *nlme* library.

Seasonal patterns of abundance for each species were examined by plotting the monthly average (over reefs) best linear unbiased predictions (BLUPs) [29] corresponding to the intra-annual effects.

In addition to the nested model above, separate models were fitted to subsets of the data corresponding to each temporal scale (e.g., only the instantaneous or the daily replicates), to evaluate the sensitivity of the estimates of spatial variation.

Fish counts were log-transformed to obtain additive effects. Due to the absence of some species in a few sampling occasions, we added a constant ( = 1) to all raw fish counts [ln(y+1)], except in the case of *P. semifasciata,* whose counts were always positive. In the latter, counts were standardized as number of individuals per 25 m of transect length, because the three reefs surveyed had different lengths. We excluded a few reefs considered as marginal non-representative habitat in the cases of *S. oculatus* and *N. bergi*, where the mean abundance over all censuses was less than one fish per transect.

### Coefficient of variation vs. mean counts

The relationship between the empirical CVs and mean fish counts was examined for the instantaneous, daily, weekly and monthly scales. Percent CVs were calculated from the raw counts for each species and transect, by selecting the censuses replicated at each temporal scale.

## Results

### Diurnal and tidal variation

Fish abundance did not vary significantly with time-of-day or tidal state; the inclusion of the fixed effect in the models did not reduce the residual variation significantly (Table 1). The exception was *P. brasilianus*, for which counts were significantly higher at low tides (p = 0.034) (Table 1). This effect, however, was not consistent across reefs: while in eight transects abundance was a 44% higher on average at low tide, in the remaining four it was stable or decreased slightly (mean = −8%). These differences were unrelated to the depth of the reefs.

**Table 1:** Effects of Time-of-day and Tide on fish counts.

					Spatial variation	Residual variation
Species	Data set	Transects	Model	Fixed effect	SD	SD
***A. patachonicus***	1	12	**Time-of-day**	0.005 (ns)	0.70	0.13
			**Null**	-	0.70	0.12
	2	12	**Tide**	0.088 (ns)	0.84	0.14
			**Null**	-	0.84	0.15
***S. oculatus***	1	8	**Time-of-day**	0.252 (ns)	0.88	0.26
			**Null**	-	0.88	0.30
	2	8	**Tide**	0.047 (ns)	1.32	0.30
			**Null**	-	1.32	0.27
***P. brasilianus***	1	12	**Time-of-day**	0.005 (ns)	0.37	0.19
			**Null**	-	0.37	0.18
	2	12	**Tide**	0.183 (*)	0.36	0.19
			**Null**	-	0.35	0.22
***N. bergi***	1	6	**Time-of-day**	0.097 (ns)	1.26	0.92
			**Null**	-	1.29	0.84
	2	9	**Tide**	0.433 (ns)	1.10	0.73
			**Null**	-	1.07	0.75

Fixed-effect coefficients for levels afternoon and high-tide, and their significance (in parenthesis), and standard deviation (SD) of spatial and residual components of variability. The number of replicates (i.e., number of reefs censused) for each data set is indicated in the “Transects” column. Significance is indicated as: ns = not significant (p>0.05), *  =  significant (p<0.05), **  =  highly significant (p<0.01). Spatial variation was highly significant in all cases.

### Temporal variation

**Figure 2:**
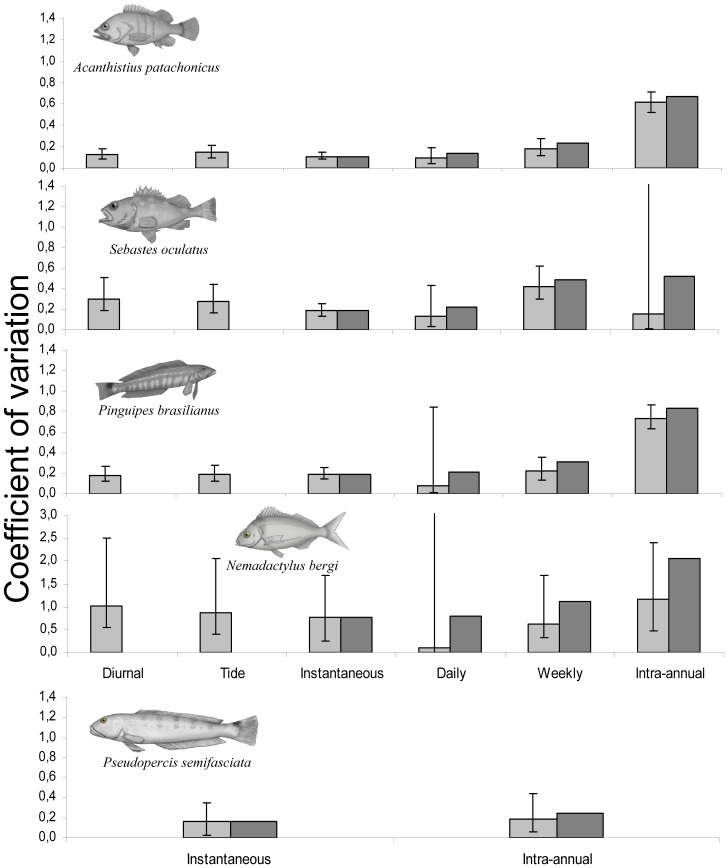
Temporal components of variability in counts. Coefficients of variation associated with different factors and temporal scales of variability in fish counts, estimated using mixed- and random-effect models fitted to census data for five reef-fish species. Soft grey bars represent the component of variation contributed by each temporal scale (with 95% confidence intervals) and darker grey bars represent the total (cumulative) variation of counts at each temporal scale. Note the different scale of the y-axis for *Nemadactylus bergi*.

**Figure 3:**
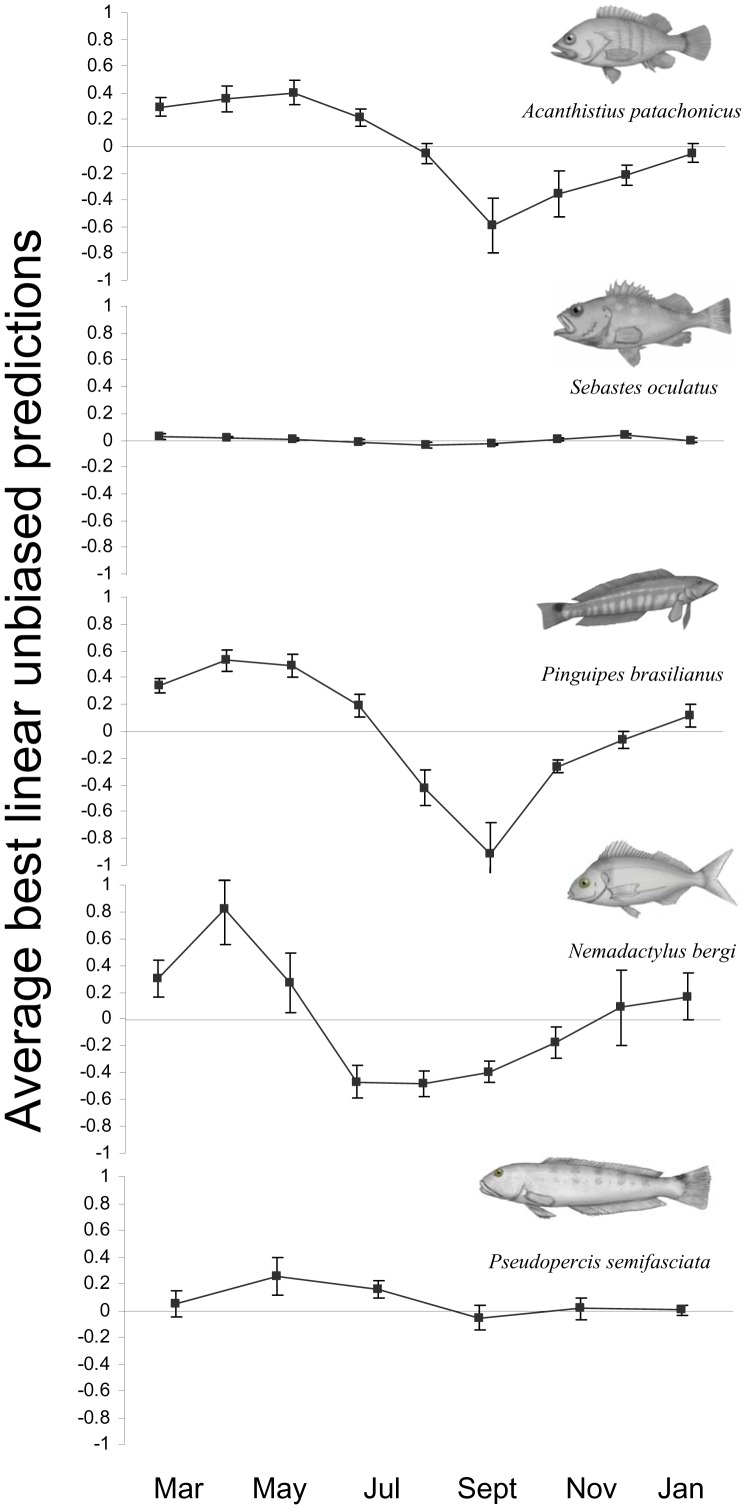
Seasonal patterns of abundance for the five rocky reef species. Monthly average (over reefs) best linear unbiased predictions (BLUPs) corresponding to the intra-annual effects ± one standard error are shown for each species.

Daily and weekly components of variability in fish counts were in general of similar magnitude to the instantaneous variation. A remarkably low daily variation (with broad 95% confidence bounds) was estimated for *N. bergi* and *P. brasilianus* (Fig. 2). In all cases, the contribution of the daily component to the overall variability was not statistically significant, while that of the weekly scale was significant for *A. patachonicus*, *S. oculatus* and *P. brasilianus* (Table 2). The intra-annual component showed the highest CV for most species, indicating strong seasonal patterns in fish abundance; the contribution of the intra-annual variation was statistically significant, except for *S. oculatus* and *P. semifasciata*, whose densities remained rather stable throughout the year (Table 2; Figs. 2 and 3).

**Table 2:** Spatial and temporal components of variability.

		Temporal variation				Spatial variation			
Species	Habit	Sources	p	SD	%VC	Transects	p	SD	%VC
***A. patachonicus***	**Sedentary**	**Total**	-	0.61	39.70	12	***	0.75	60.30
		**Instantaneous**	-	0.11	1.25				
		**Daily**	ns	0.09	0.95				
		**Weekly**	**	0.18	3.40				
		**Intra-annual**	***	0.56	34.10				
***S. oculatus***	**Sedentary**	**Total**	-	0.49	11.02	8	***	1.40	88.95
		**Instantaneous**	-	0.18	1.52				
		**Daily**	ns	0.13	0.78				
		**weekly**	***	0.41	7.69				
		**Intra-annual**	ns	0.15	1.03				
***P. brasilianus***	**Sedentary**	**Total**	-	0.72	73.11	12	***	0.44	26.70
		**Instantaneous**	-	0.19	5.17				
		**Daily**	ns	0.08	0.95				
		**Weekly**	*	0.22	6.67				
		**Intra-annual**	***	0.66	60.32				
***P. semifasciata***	**Sedentary**	**Total**	-	0.24	86.70	3	***	0.10	13.30
		**Instantaneous**	-	0.16	36.10				
		**Intra-annual**	***	0.18	48.20				
		**Inter-annual**	***	0.04	2.40				
***N. bergi***	**Schooling**	**Total**	-	1.29	85.76	11	***	0.53	14.20
		**Instantaneous**	-	0.69	24.27				
		**Daily**	ns	0.09	0.46				
		**weekly**	ns	0.57	16.93				
		**Intra-annual**	***	0.93	44.10				

Standard deviations (SDs) of spatial and temporal components of variability, and their percent contribution (%VC) to total variance for each species. The number of replicates (i.e., number of reefs censused) for each species is indicated in the “Transects” column. The significance of the variance components is indicated in the column denoted with “p”: ns =  not significant (p>0.05), *  =  significant (p<0.05), **  =  highly significant (p<0.01), ***  =  very highly significant (p<0.001). The instantaneous variation was estimated as the residual error of the nested random models; therefore its significance was not evaluated.

The temporal variation in counts of *A. patachonicus* and *P. brasilianus* was relatively small at the instantaneous, daily and weekly scales, with respective 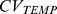 of 0.11, 0.09 and 0.18 in *A. patachonicus*, and 0.19, 0.08 and 0.22 in *P. brasilianus*, much lower than those of the intra-annual component (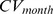 of 0.56 and 0.66 for the two species). The total contribution of the short time scales (i.e., instantaneous, daily and weekly) to the overall variability was 5.6% in *A. patachonicus* and 12.8% in *P. brasilianus,* compared to 34.1% and 60.3% due to the intra-annual component.

The temporal variation in *S. oculatus* counts was relatively small at all time scales with the exception of the weekly scale, which had the largest contribution (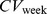 = 0.41, compared to 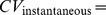
0.18, 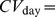 0.13 and 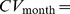
0.15). The contribution of the monthly scale was not significant (Table 2), although shallower reefs presented a slight seasonal pattern, with a winter drop in abundance. Similar to *S. oculatus*, repeated and intra-annual counts of *P. semifasciata* had relatively low and similar *CV*s (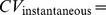
0.16 and 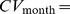
0.18) (Table 2; Figs. 2 and 3).

Temporal variation of *N. bergi* counts was higher than that observed for sedentary species, although the daily and weekly contributions were not statistically significant (Table 2; Fig. 2). Fish counts were dominated by a strong seasonal component: this species was virtually absent in winter and spring (Table 2; Fig. 3).

### Spatial heterogeneity

**Figure 4:**
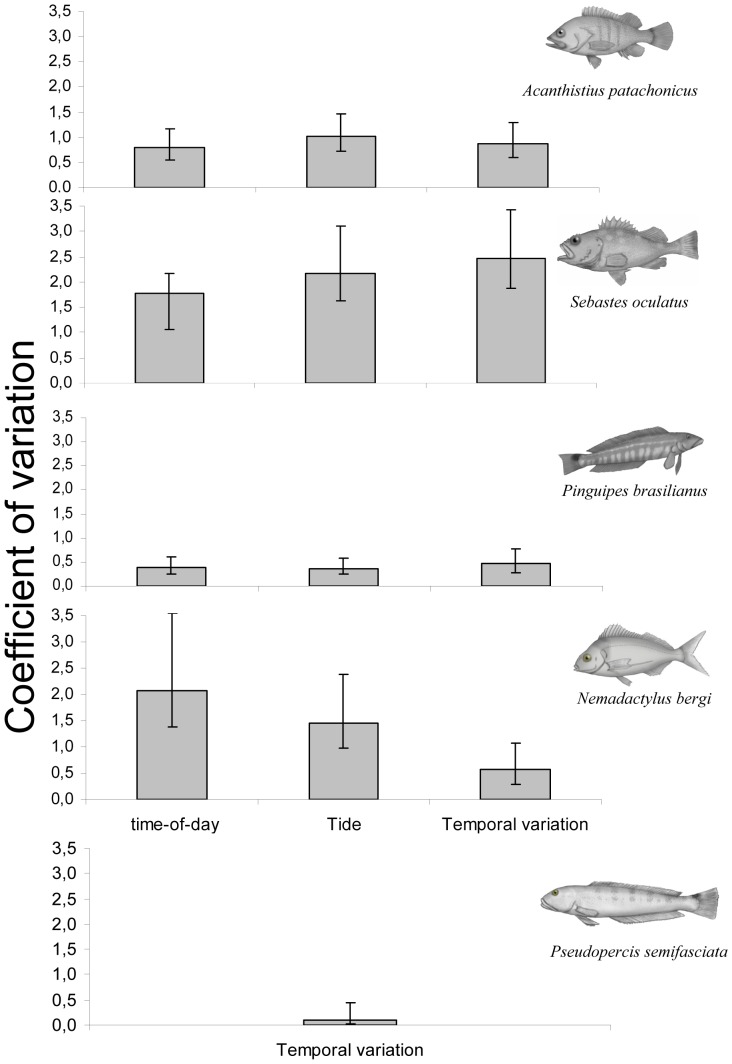
Spatial coefficients of variation in counts. Coefficients of spatial variation in fish counts estimated by the mixed-effect models used to evaluate the effects of time-of-day and tidal level, and by the random-effect models used to estimate variability at different temporal scales.

When separate models were fitted to subsets of the data corresponding to each temporal scale (e.g., only the instantaneous or the daily replicates), the estimated spatial variation in fish counts was similar across temporal scales and factors analyzed, for all the species studied. This supported the assumption made in the nested model that the spatial variability was time-invariant. In the case of *N. bergi*, however, the
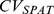
estimated from the mixed (Table 1) and random models (Table 2) are not directly comparable because different transects were used for model fitting. *Sebastes oculatus* showed the largest spatial variability (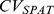 between 1.08 and 2.47) (Tables 1 and 2; Fig. 4), with fish counts ranging from 0 to up to ∼200 individuals per transect. In both *S. oculatus* and *A. patachonicus* spatial variability was larger than temporal variability, amounting to 94% and 60% of the total variance, respectively. On the other extreme, *P. semifasciata* and *P. brasilianus* showed the smallest spatial variation; their counts per 25 m of linear reef ledge had the narrowest range: between 1 and 5 for *P. semifasciata,* and between 5 and 45 for *P. brasilianus* (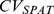 equal to 0.10 and 0.44, respectively) (Fig. 4).

### Coefficient of variation vs. mean

**Figure 5:**
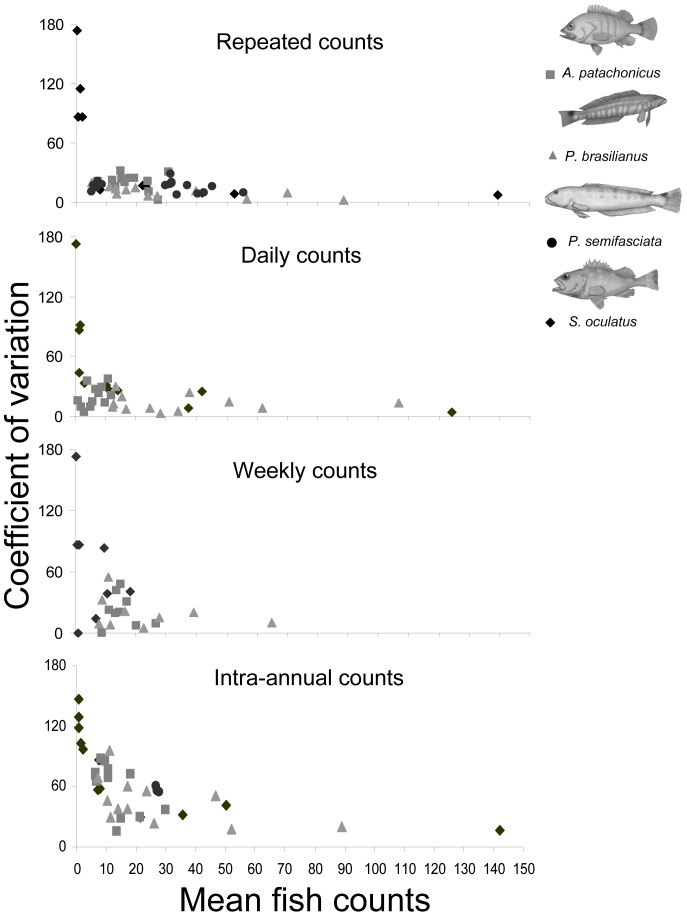
Coefficients of variation versus mean fish counts. Empirical coefficients of variation versus mean fish counts for each temporal scale studied. Each point corresponds to one transect or reef.

The CVs of the raw fish counts for sedentary species were inversely related to their mean abundance (Fig. 5). Although there were several cases in which both were low, high mean abundance always had low CVs. This relationship was consistent for all the temporal scales considered: all showed a similar pattern while the level of variability increased with time elapsed between censuses. *Nemadactylus bergi* was excluded because its much larger CVs and few records did not allow detecting any clear pattern.

## Discussion

Our results showed that instantaneous variation represents a non-negligible (>10%) basal level of variability that should be taken into account in the design of monitoring programs, or when interpreting past visual census estimates of reef fish abundance in northern Patagonia. McClanahan et al. [14] drew attention to this important and often ignored source of variation, which can lead to difficulties in detecting differences over time and between sampling methods or sites (e.g., in response to management regulations). In their case, the estimates of instantaneous variation could be somewhat inflated by the inclusion of between-diver differences.

The most relevant feature of our approach is that it allowed a direct comparison between the magnitude of the different sources of variability –both spatial and temporal– that affected fish counts, by incorporating them into random-effect models. An advantage of this method is that it can be applied to unbalanced designs such as the one used in this study. This is not the case of standard methods used for variance decomposition based on analysis of variance, which do require that the sampling design be balanced [3], [32], [33].

### Diurnal and tidal variation

In our comparisons, fish counts did not differ significantly between morning and afternoon, similarly to other studies [4], [16], [34], [35]. Nor could we detect an effect of tidal state for most species, a result that is also consistent with previous works [34], [36]. The only exception was *P. brasilianus*, which was more abundant at low tide in some of the reefs (Table 1). This species is the only among the species studied that occupies the intertidal zone during high tide (AJI, personal observation), suggesting that some fish could move between subtidal and intertidal rocky areas following the tidal cycle, and that fish abundance may increase in subtidal shallow reefs during the low tide.

Most previous studies did not consider tidal state explicitly in their sampling designs, thus any potential effects of tide may add “noise” or be confounded with those of other factors. Although we did not find a strong influence of tide on our comparisons, its effects were evaluated based on censuses made on a single day; therefore possible interactions with other factors (e.g., season, tidal amplitude, spawning activity) could not be evaluated. This limitation is particularly relevant because tide is a complex factor, correlated with other variables such as current speed and direction, depth and availability of suitable intertidal habitats. Furthermore, as current speeds are highly influenced by bottom topography, the extrapolation of our results to other sites in relation to the effect of tide would not be appropriate. Fish movements in response to currents are well known by fishers, and they were demonstrated for other reef species [37].

### Temporal variation in fish counts

Compared to UVCs conducted in warmer regions, the variability in fish counts estimated in this study was low for sedentary species, but similar for schooling species [4], [14], [17], [38]. Among time scales shorter than a year, the contribution of the instantaneous variation was in general similar to that of the other short temporal scales (i.e., daily and weekly). Variability in counts between low and high tides, and between morning and afternoon, was similar in magnitude to the instantaneous variation (i.e., the residual error in the nested random models), reinforcing the conclusion that differences in counts could be due to instantaneous stochastic variation, similar to the results reported by McClanahan et al. [14]. In the case of *P. semifasciata*, the repeated counts were conducted by different divers, which may have inflated the estimates of instantaneous variation, even though the diver effect was found to be non significant [24]. Similarly, as tidal state was not controlled in the sampling design of the temporal censuses, it may have contributed to the variability estimated for *P. brasilianus*. Except for *P. semifasciata* and *S. oculatus*, the other species analyzed (*A. patachonicus*, *P. brasilianus* and *N. bergi*) showed high intra-annual variation associated with a strong seasonal pattern (Fig. 3). Abundance trends for these species paralleled the annual cycle of water temperature in Nuevo Gulf: fish abundance increased slowly during spring and summer, peaked in autumn when water temperature is maximum (17°C in March-April), and diminished gradually towards late winter, when the water is coldest (9°C in August-September). This trend was much more marked for *N. bergi*, which was virtually absent during winter (for more details on the seasonal patterns of abundance see [24], [39]).

The levels of variability estimated for the different species were consistent with their life history attributes and behavior. The instantaneous variability was smallest for *A. patachonicus*, followed by *P. semifasciata*, *S. oculatus, P. brasilianus* and *N. bergi*, in that order. This ranking can be mostly explained in terms of degree of site-attachment and swimming speed. *Acanthistius patachonicus*, for example, is a sedentary species commonly observed in close proximity (within 5 m) to the refuge [40], which facilitates censusing. This makes *A. patachonicus* a reliable candidate for assessing the status of the reefs and the impact of angling and spear-fishing, or even for detecting small changes in abundance. *Pinguipes brasilianus* and *P. semifasciata* are more mobile than *A. patachonicus*. While these species can be observed resting on the bottom on their pelvic fins, in general they swim in and out of the refuges and of the area censused. For example, one individual of *P. semifasciata* was spotted in places distant up to 46 m along a reef ledge over a 1-hour period (although most, i.e., 30 of 35 fish, remained within 13 m) (LAV, unpublished data), and it is common to observe fish more than 20 m far from the reefs feeding on soft bottoms (AJI, personal observations).

The schooling species *N. bergi* had the highest variability at all temporal and spatial scales due to high mobility and variation in group size. Similar patterns have been found for others schooling species (e.g., Lutjanidae, Acanthuridae and Scaridae families [4], [14], [25]). When counting these species, observation events regularly record fish schools as a unit, whilst in sedentary species they record individual fish. This explains the high variability observed, as the presence or absence of a school could easily result in counts changing from 0 to 100 individuals. The information provided by visual censuses on schooling species would be limited to detecting their presence and seasonal trends in the reefs.

### Spatial variation

The design of impact studies should address the spatial heterogeneity in order to avoid confounding its effects with the factors under investigation [3]. Our results show that patterns of spatial heterogeneity differed among species, a result of the complex interactions between morphological, physical and ecological characteristics of the reefs, and the particular microhabitat and food requirements of each of the species studied. Amongst the sedentary species, *S. oculatus* showed the highest spatial variation due to marked contrasts in abundance between shallow and deep reefs. Conversely, *P. brasilianus*, a pinguipedid with an apparently weak microhabitat association [39], had low spatial variation (Fig. 2). In the case of *P. semifasciata*, reefs with high fish abundance were intentionally selected to investigate seasonal patterns [24]; hence their spatial variation (the lowest estimated) is not representative of the variability corresponding to a random collection of reefs. Overall, spatial heterogeneity for each species was consistent across the different temporal scales and/or factors analyzed; thus environmental differences between reefs could be considered stable between seasons.

### Implications for design of monitoring programs

The instantaneous variation, understood as baseline variation or error in fish counts, tends to obscure relevant patterns at larger temporal and/or spatial scales. Its magnitude sets the minimum variance to be used in power analyses to calculate minimum sample sizes required for detecting any given change in abundance. Given that instantaneous variation and detectability are strongly related to swimming behaviour, shape and size ([4], [9], [14]–[16] and our results), and are independent of spatial heterogeneity [16], it is possible to use published results for other systems and similar species to explore power and sample size requirements. In the case of schooling species, for example, only large effects could be detected given the high variability of UVC counts at all scales.

In general, the power of UVC experiments may be increased by augmenting either the number of replicates or the length of the transects. The level of sampling effort would of course depend on a trade-off between the desired precision and logistics and monetary constrains. Increasing the number of replicates per transect would increase the statistical power by reducing the variance of the average in proportion to 1/n. Faster reductions may be possible by increasing the length of the transects in cases in which instantaneous variability is caused not so much by counting errors but by fish moving along a reef ledge, in and out of the sampled portion. This is the case of *P. semifasciata* and *P. brasilianus*. In such cases, the best alternative, for any given total distance covered, would be to use longer transects rather than to replicate short transects. Overall, the choice between increasing the number of replicates or the length of the transects should depend on the size and shape of the fishes' home ranges, and on their degree of association with specific portions of the reefs. Longer transects would be required in studies focused on scarce or rare species, given the inverse relationship between variability and mean fish counts found for all sedentary species at all temporal scales (Fig. 5). This relationship is consistent with previous studies [4], [14].

Some studies have found that sampling sites and instantaneous variation in counts made by a single diver were more important sources of variation than diver identity [14]–[16]. On this basis, the authors proposed that a better choice to increase power would be to increase sampling effort using the same group of divers rather than spending valuable water time to undertake prolonged diver retraining [16]. Diver inter-calibration and training would become important for longer-term studies in which divers change over time [16]. When the focus is on temporal comparisons, our results, in concordance with those of Thompson & Mapstone [4], suggest that the use of fixed transects is a good strategy to increase power, and to remove the spatial heterogeneity by treating the sampling units as a random effect. In most cases, high between-site variability could obscure most temporal patterns if not accounted for. Additionally, for inter-annual comparisons or in short time-scale experiments, UVCs should be done preferentially during periods of highest abundance, when CVs are lower.

Regarding diurnal variation, our results agree with the recommendation of Thompson and Mapstone [4], that sampling during the middle of the day (between 2 to 3 h after sunrise and 2 to 3 h before sunset) is a good strategy to minimize possible time-of-day effects. It is noteworthy that time-of-day effects are generally associated to crepuscular periods [41], [42], not investigated in this study. Finally, tidal state could not be discarded as a factor that may affect abundance estimates; fixing the tidal state on sampling designs or evaluating the tidal effects on experiments or monitoring programs appears to be necessary.

## Supporting Information

Text S1 **Details of sampling design.** Dates and sampling hours for time-of-day and tide experiments.
(DOC)

Text S2 **Statistical models.** Hierarchical random-effect model used to estimate variance components for each species.
(DOC)
