# Supplementary material for: Variability in Abundance of Temperate Reef Fishes Estimated by Visual Census
Source: PLoS One. 2013 Apr 4;8(4):e61072. doi: 10.1371/journal.pone.0061072 (PMC3617182; doi:10.1371/journal.pone.0061072)
Supplement: Text S1 — Details of sampling design. Dates and sampling hours for time-of-day and tide experiments. (DOC) [file pone.0061072.s001.doc]

**Supplementary Text S1**

**Details of sampling design**

To estimate the effect of time-of-day, the sampling date of 29 March 2007 was selected because low tide occurred at 1314 hrs; hence the morning and afternoon censuses would be both conducted at approximately the same tidal height. In that case, all transects were surveyed twice: in the morning between 0955 hrs and 1111 hrs, and again in the afternoon, between 1513 hrs and 1734 hrs.

The date 5 May 2007 was selected to conduct these censuses because the low tide occurred at 1032 hrs and the high tide at 1620 hrs (the censuses were conducted between 0951 hrs and 1142 hrs, and between 1528 hrs and 1709 hrs).
