# Supplementary material for: Variability in Abundance of Temperate Reef Fishes Estimated by Visual Census
Source: PLoS One. 2013 Apr 4;8(4):e61072. doi: 10.1371/journal.pone.0061072 (PMC3617182; doi:10.1371/journal.pone.0061072)
Supplement: Text S2 — Statistical models. Hierarchical random-effect model used to estimate variance components for each species. (DOC) [file pone.0061072.s002.doc]

**Supplementary Text S2**

**Statistical models**

For each species, the hierarchical random-effect model used to estimate variance components was constructed as:

*y_rmwdi_ = μ + β_r_ + δ_rm_ + γ_rmw_ + λ_rmwd_ + ε_rmwdi_*

where:

*y_rmwdi_* = log of the number of fish + 1 counted in the *i*th census of the *r*th reef, conducted in day *d*, week *w*, and month *m*;

*μ* = overall mean;

*β_r_* = effect of the *r*th reef;

*δ_rm_* = effect of the *m*th month (i.e. intra-annual variation) in the *r*th reef;

*γ_rmw_* = effect of the *w*th week (i.e. weekly variation), within the *m*th month in the *r*th reef;

*λ_rmwd_* = effect of the *d*th day (i.e. daily variation), within the *w*th week, within the *m*th month in the *r*th reef; and

*ε_rmwdi_* = residual error corresponding to instantaneous variation.

In the case of *P. semifasciata* a year factor was added to account for inter-annual differences in abundance. All random effects were assumed to be normally distributed with mean zero and respective variances$\sigma_{\beta}^{2}$, $\sigma_{\delta}^{2}$, $\sigma_{\gamma}^{2}$, $\sigma_{\lambda}^{2}$ and $\sigma_{\varepsilon}^{2}$.
